# Optimization of Carob Products Preparation for Targeted LC-MS/MS Metabolomics Analysis

**DOI:** 10.3390/metabo13050645

**Published:** 2023-05-09

**Authors:** Olga Deda, Olga Begou, Helen Gika, Georgios Theodoridis, Agapios Agapiou

**Affiliations:** 1 Laboratory of Forensic Medicine and Toxicology, School of Medicine, Aristotle University of Thessaloniki, 54124 Thessaloniki, Greece; 2 Biomic Auth, Bioanalysis and Omics Laboratory, Centre for Interdisciplinary Research of Aristotle University of Thessaloniki, Innovation Area of Thessaloniki, 57001 Thermi, Greece; 3 Department of Chemistry, Aristotle University of Thessaloniki, 54124 Thessaloniki, Greece; 4 Department of Chemistry, University of Cyprus, P.O. Box 20537, Nicosia 1678, Cyprus; agapiou.agapios@ucy.ac.cy

**Keywords:** HILIC-MS/MS, bioactive compounds, metabolites, sample preparation, optimization, amino acids

## Abstract

Carob (*Ceratonia siliqua*) is an exceptional source of significant bioactive compounds with great economic importance in the Mediterranean region, where it is widely cultivated. Carob fruit is used for the production of a variety of products and commodities such as powder, syrup, coffee, flour, cakes, and beverages. There is growing evidence of the beneficial effects of carob and the products made from it on a range of health problems. Therefore, metabolomics could be used to explore the nutrient-rich compounds of carob. Sample preparation is a crucial step in metabolomics-based analysis and has a great impact on the quality of the data obtained. Herein, sample preparation of carob syrup and powder was optimized, to enable highly efficient metabolomics-based HILIC-MS/MS analysis. Pooled powder and syrup samples were extracted under different conditions by adjusting pH, solvent type, and sample weight to solvent volume ratio (Wc/Vs). The metabolomics profiles obtained were evaluated using the established criteria of total area and number of maxima. It was observed that the Wc/Vs ratio of 1:2 resulted in the highest number of metabolites, regardless of solvent type or pH. Aqueous acetonitrile with a Wc/Vs ratio of 1:2 satisfied all established criteria for both carob syrup and powder samples. However, when the pH was adjusted, basic aqueous propanol 1:2 Wc/Vs and acidic aqueous acetonitrile 1:2 Wc/Vs provided the best results for syrup and powder, respectively. We strongly believe that the current study could support the standardization of the metabolomics sample preparation process to enable more efficient LC-MS/MS carob analysis.

## 1. Introduction

The carob tree has been cultivated in the Mediterranean region for centuries, making an important contribution to the society and economy of many European (e.g., Spain, Italy, Portugal, Greece, Cyprus, etc.) and Middle Eastern countries (e.g., Egypt, Tunisia, Morocco, etc.). Its popularity is mainly due to its excellent nutritional and medicinal properties, as it is considered a functional food associated with the Mediterranean diet [1]. In the last decade, the interest in the sustainability and valorization of the carob tree (fruits, pods, and leaves) has increased beyond its unique agricultural importance as a fire- and drought-resistant tree [2]. Therefore, in modern societies, where the circular economy and climate change are gaining emerging attention, the potential contribution of the carob tree to the United Nations Sustainable Development Goals (UN SDGs) is driven by the promotion of social (no poverty), health (well-being), and ecological (restoration and support of terrestrial ecosystems) balance; as a nutraceutical product, it supports UN SDGs 1–3, 5, 12, and 15 [3]. 

The chemical composition of the carob fruit depends on multiple factors such as cultivar, origin, climate conditions and harvesting time. Carob contains high amount of sugars (mostly sucrose, glucose, and fructose), proteins, minerals, and dietary fibers. It is also rich in polyphenols, such as tannins, flavonoids, and phenolic acids, and contains lipids and vitamins, but is a caffeine- and theobromine-free pod with low fat content [4,5].

In analytical chemistry, there is a growing body of research on the importance of sample preparation, perhaps the most intensive, multi-stepped, time-consuming, and costly stage, before chemical analysis. As reported in Table 1, analysis of carob and its products includes the use of single, hyphenated, and instrumental platforms with low or advanced detection capabilities, covering mainly the fields of chromatography (liquid and gas chromatography), spectrometry (LC-MS, GC-MS, and tandem MS/MS) with low (photodiode array (PDA), refractive index (RI)) or high resolution (HR) detectors, and spectroscopy (NMR). As detailed below, almost all parts of the carob tree have been analysed for their phytochemical constituents. The nutritional benefits of carob pods, and traditional products of Cyprus origin are presented in [6]. The small molecules, called volatile organic compounds (VOCs), are responsible for the intense flavoring power of carob pods [7] and can be correlated with carob’s ripening [8]. VOCs in association with multivariate data analysis (MDA), achieved the discrimination of carob pods based on their geographical origin [9]; the same was performed using MDA and carob’s nutritional compositional values [10].

Lately, a wide range of sample extraction techniques have been applied to achieve better extraction performance from food matrices in general, and in particular for carob matrix: maceration (ME), liquid-liquid extraction (LLE), solid-phase extraction (SPE), pulsed or continuous ultrasound-assisted extraction (UAE), microwave-assisted extraction (MAE), Soxhlet extraction, supercritical fluid extraction (SFE), QuEChERS, etc. All approaches aim to improve the selectivity and sensitivity of analytical methods, while boosting green extraction techniques with the goals of protection of the operator and the environment by minimizing solvent use [11]. For example, the phenolic content and antioxidant capacity of carob pods varied significantly with type of extraction process (UAE, MAE, Soxhlet, and SFE-CO_2_ were investigated) [12]. The work of Christou et al. (2021) emphasized the importance of UAE parameters of solid-solvent ratio, solvent concentration, extraction time, sonication amplitude, and sonication mode in the extraction of carob polyphenols [13]. Huma et al. (2018) focused on the MAE technique and its optimal conditions: microwave power, ethanol concentration, and solvent-to-sample ratio were tested for the extraction of total phenolic content (TPC) and condensed tannins [14]. Other researchers used the extracts of different solvents (diethyl-ether and ethyl acetate) to highlight the anti-cancer activity of carob pods [15]. Overall, a wide variation is observed in the applied extraction techniques, the employed analytical tools, as well as the agricultural characteristics of the investigated carob samples (e.g., carob variety, geographical origin, cultivation practices, maturation, altitude, micro-environment, etc.) [16]. This creates variations in the published results both on qualitative and quantitative level. 

The simultaneous analysis of several endogenous compounds in a single analytical run can present challenges due to the different physicochemical properties of analytes of interest. Hydrophilic interaction liquid chromatography (HILIC), suitable for the analysis of polar or/and semi-polar metabolites, has been employed as a complementary chromatographic technique to reverse phase (RP) chromatography or ion-pair chromatography (IPC) for MS analysis. Commonly, in HILIC mode chromatography, a polar stationary phase is used (e.g., non-silica-based amino packings, underivatized silica, or modified silica), combined with high organic content (>90% *v*/*v*) mobile phases with the addition of different additives, such as HCOONH_4_ or CH_3_COONH_4_ [17]. 

The most important but tedious and difficult part of the analysis is that of sample preparation. According to a recent study by Justyna Potka-Wasylka et al. (2022), nearly 80% of the workflow time for instrumental chemical analysis is spent on sample preparation before analysis, a difficult and time-consuming step that directly affects the quality of the results even though operators occasionally underestimate this step [18]. The present work aims to determine the optimal protocol for the analysis of two carob matrices, namely syrup (liquid) and powder (solid), in order to identify the largest number of metabolites; towards this, the effects of extraction, pH adjustment, solvent, and sample weight to solvent volume ratio (Wc/Vs) were examined. To our knowledge, there is no other work on sample optimization of carob products using HILIC-MS/MS.

**Table 1:** Reported analytical methods for carob and carob byproducts profiling and characterization.

Concept	Substrate	Method	Sample Preparation	Number and Chemical Classes of Detected Compounds	Ref.
Roasting	Carob beans from Egypt, unroasted & roasted pods	HS-SPME-GC/MS	100 mg carob pod + IS ((Z)-3-hexenyl acetate), 30 min at 50 °C with the SPME fiber	31 volatile compounds: short chain fatty acids, aldehydes, acids, alcohols, aldehydes/ketones, esters, furans/pyrans, sesquiterpenes, hydrocarbons	[19]
Different geographical origin, ripening stage and roasting process	Pods from different locations in Egypt Different ripening stages (unripe green, mid ripe to full ripe)	GC-MS and UHPLC-ESI-HR-MS/PDA	18 mg dried fruit powder homogenization in 1.2 mL MeOH + IS (umbelliferon) + ultrasonic bath for 20 min + vortex + centrifugation + solid phase extraction (SPE), elution with MeOH + N_2_ evaporation to dryness + reconstitution to MeOH	70 primary metabolites: carbohydrates (mono- and di-saccharides), phenolic acids, organic acids (and also amino acids), fatty acids, sterols, and nitrogenous compounds 83 compounds: flavonoids, fatty acids, phenolic acids, tannins, and carbohydrate derivatives	[20]
Bioactive properties	Carob seeds Tunisian locust bean seeds, pods	HPLC-FLD (fluorescence detector) and LC-DAD-ESI/MSn	1.5 g dried powder extracted in 25 mL of metaphosphoric acid, and placed under magnetic stirring (150 rpm) for 20 min + filtration. Extraction in carob seeds were performed by maceration (ME) and ultrasound-assisted extraction (UAE). For both extraction methods, water and ethanol were chosen as solvents, with four different proportions used: (i) EtOH:H_2_O (25:75; *v*/*v*); (ii) EtOH:H_2_O (50:50; *v*/*v*); (iii) EtOH:H_2_O (75:25; *v*/*v*); and (iv) 100% H_2_O For ME, the dried powdered samples (1 g) were placed in a beaker with 30 mL of each of the four solvents, under magnetic stirring 150 rpm for 1 h at room temperature + filtration and the extraction procedure were repeated with an additional portion of the solvent. The obtained extracts were combined, the EtOH was removed, and the residual aqueous phase was frozen and lyophilized. The UAE was carried out in an ultrasonic device: 3 g dried powdered samples extracted with 100 mL of each of the four solvents by the ultrasonic device at 375 W for 10 min + filtration and, as for the ME, the EtOH was removed, and the residual aqueous phase was frozen and lyophilized.	Tocopherols, organic acids	[21]
Ripening	Carob fruits	Soluble carbohydrates: HPLC-RI Macro-minerals: ion chromatography coupled to conductivity detector (IC-ConD) Polyphenols: UHPLC-Q-Orbitrap-HRMS	Intact carob fruits were frozen at −40 °C. Lyophilization at 0 °C for 48 h. Blender lyophilization for about 24 h Cyclotech mill 0.3 g of freeze-dried carob pulp extracted with 10 mL mixture of EtOH:H_2_O (80:20, *v*/*v*) + vortex + sonication + centrifugation + filtration	Polyphenols, catechins, tannins	[22]
Flavonoid content in leaf (carob among other)		HPLC-MS	Extraction of oven-dried leaves with 70% EtOH + evaporation 0.5 g from each extract dissolved in 14 mL H_2_O:EtOH (20:80)	22 flavonoids	[23]
Antioxidant activities of two commercial carob flours	Commercial carob flours	NMR & HPLC	Enzymes of carob flours (1 g) were inactivated by boiling in H_2_O for 5 min. The slush was filtered through ten layers of gauze and the resultant liquid adjusted to pH 6.0 with NaOH, and then lyophilized. 1 g of dry mass extracted with 10 mL of boiling water for 5 min + centrifugation + filtration	Dietary fiber, total phenols, pinitol and antioxidant activity	[24]
Comparison of the sugar levels in wheat flour and wholemeal wheat flour plant-based high-protein ingredients, e.g., carob high-protein ingredients (HPIs)	Wheat flour and wholemeal wheat flour	HPLC-RID (refractive index detector)	Test of different extraction procedures: Six are based on aqueous extraction and three are based on ethanolic extraction. A: 2 g of samples diluted in 8 mL H_2_O + vortex mixed + 20 min shaking + centrifugation. Additional dilution of the supernatant with 10 mL of H_2_O + filtration. B: 2 g of samples diluted in 15 mL EtOH 80% + vortex + sonication + centrifugation. Two-step extraction with addition of 15 mL of EtOH 80% + vortex+ sonication. Evaporation of the pooled supernatants. reconstitution with water + filtration	Short-chain carbohydrates Ethanolic extraction was chosen	[25]
Phenolic content of extracts derived from Cypriot carob pods using different solvents	Whole fruit (pulp + seeds) and extracts given to cells	LC-MS	DE, EA, EtOH and H_2_O as solvents DE and EA were more effective	Polyphenols found in EA and DE ripe pulp and seeds carob extracts: apigenin, myricetin, rutin, naringenin, ferulic acid, kaempferol and gallic acid	[15]
Detection and formation of D-Amino acids in processed plant saps, syrups, and fruit juice	Saps and juices of trees (maple, palm, birch), fruits (grape, apple, pear, pomegranate, date), and various other plants (agave, beetroot, sugar cane, carob)	Enantioselective GC-MS	Samples of 1 g were diluted with 5 mL H_2_O and adjusted to pH 2.3 (0.01 M HCl) + SPE, elution with 4 M aqueous ammonia (5 mL) + evaporation to dryness + 0.1 M HCl (0.5 mL) + evaporation to dryness + 500 µL of acetyl chloride in 2-propanol + 1 h at 100 °C + evaporation + 500 μL DCM and 100 μL pentafluoropropionic anhydride + 20 min at 100 °C + evaporation to dryness + reconstitution with 500 μL DCM	Saccharides (glucose, fructose, or sucrose) and containing amino acids	[26]
Phenolic compounds in wood of *Ceratonia siliqua*	Wood of *Ceratonia siliqua* (carob)	GC-MS	Sample of 1 g extracted with MeOH:H_2_O + evaporation. The aqueous phase extracted with PE (2 × 25 mL), then with DE (2 × 25 mL), and finally with DE:MeOH (9:1; 2 × 25 mL). For hydrolysis, aqueous extract was mixed with 6 mL MeOH:H_2_O HCl (6 m; 1:1) + oven-heated at 100 °C for 8 h + extraction with DE:MeOH (9:1; 2 × 25 mL) and H_2_O (2 × 25 mL) + silyllation with trimethylchlorosilane and bis-(trimethylsilyl)- trifluoracetamide (1:3).	Tannin composition	[27]
Lipid profiling in *Prosopis* spp. and *Ceratonia siliqua* seed germ flour	Flour from seed germ of European carob (*Ceratonia siliqua*)	GC-FID/MALDI-TOF	Sample of 500 mg seed germ flour (SGF) extracted with 1 mL H_2_O + 3.75 mL Chl/MeOH (1:2, *v*/*v*) + 1.25 mL Chl + 1.25 mL H_2_O + vortex + centrifugation. Re-extraction of lower phase as previously. The organic layers were combined and evaporated to dryness.	Lipids profile, fatty acids, triacylglycerols and phospholipids	[28]

Chl: Chloroform; DE: Diethyl ether; EA: ethyl acetate; EtOH: Ethanol; H_2_O: Water; HCl: Hydrochloric acid; MeOH: Methanol; PE: Petroleum ether.

## 2. Materials and Methods

### 2.1. Chemicals, Reagents and Equipment

LC-MS grade acetonitrile (ACN), methanol (MeOH), and 2-propanol (PropOH) were purchased from Sigma-Aldrich (St. Louis, MO, USA). Ammonium formate and formic acid (>99% LC-MS grade) were provided from Chem-Lab (Zedelgem, Belgium). Ammonia (28.0–30.0% NH_3_ basis) was obtained from Merck KGaA (Darmstadt, Germany). Pure water (18.2 MΩ cm^−1^) was purified in a Milli-Q device (Millipore Purification System, Merck Darmstadt, Germany).

Syringe Terumo 2.5 mL (Tokyo, Japan) was used, and PTFE filters 0.22 μm were obtained from Millex-Merk (Darmdtsdt, Germany). The Misonix XL Sonicator Ultrasonic Cell Processor (Farmingdale, NY, USA) equipment and the CyberScan 1000 (Eutech instruments PTE LTD, Singapore) pH meter were used. Vortex-mixing and centrifugation were performed on an IKA Ms1 Mini Shaker Laboratory Vortex (Staufen, Germany) and on a Micro Centaur Plus, MSE (London, UK) centrifuge, respectively. The samples included Cypriot carob powder (n = 4) and syrup (n = 6) commercial products purchased from a local market in Cyprus.

### 2.2. Sample Preparation

Pooled samples were prepared by mixing 5 g of the available carob products; four powder and six syrup, respectively. The pooled samples were vortex-mixed and divided into 40 portions of 250 mg for each matrix.

**Figure 1:**
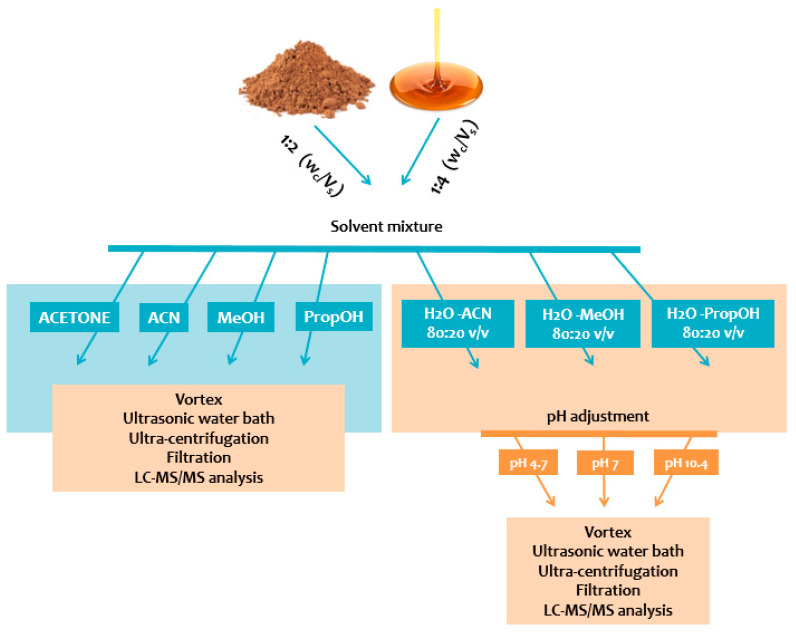
Schematic illustration of sample preparation steps.

Sample weight to solvent volume ratio (Wc/Vs), extraction solvent or solvent mixture, and pH value were evaluated. For each of the 40 aliquots (syrup or powder), the addition of 1/2 Wc/Vs and 1/4 Wc/Vs of each tested solvent or solvent mixture (acetone, ACN, MeOH, PropOH, ACN:H_2_O, MeOH:H_2_O, and PropOH:H_2_O) was performed. Only in aqueous extracts 80:20 *v/v* (ACN:H_2_O, MeOH:H_2_O, and PropOH:H_2_O), adjustment of pH value (acidic, neutral, basic) was accessed by the addition of formic acid, ammonium formate, or ammonia, respectively. The measurement of pH value was performed for all aqueous solvent-mix extracts. Every mixture was vortex-mixed for 1 min followed by sonication for 15 min and centrifugation for 20 min at 4 °C (10,000× *g*). The obtained clear supernatants were filtered through PTFE 0.22 μm syringe filters and QC samples were prepared from filtrates to evaluate the system’s analytical performance. One hundred and fifty microliters were evaporated to dryness under nitrogen stream, resuspended with 150 μL of the mobile phase, and finally transferred into 2 mL autosampler glass vials equipped with 200 μL microinserts, before being subjected to targeted LC-MS/MS analysis. The illustration of the sample preparation process is presented in Figure 1.

### 2.3. LC-MS/MS Analysis

As described in previous publications [29,30], carob extracts were analysed using a previously developed and validated targeted LC-MS/MS method performed in an ACQUITY UPLC H-Class chromatography system coupled to a Xevo TQD mass spectrometer (Waters Corporation, Milford, MA, USA), operating in both positive and negative mode [31]. The method includes 80 MRM channels for small polar metabolites. Briefly, the column was an Acquity BEH Amide (2.1 mm × 150 mm, 1.7 μm), equipped with an Acquity UPLC Van-Guard pre-column (Waters, UK). The mobile phase consisted of (a) ACN:H_2_O 95:5 *v/v* and (b) ACN:H_2_O 30:70 *v*/*v*, both containing 10 mM ammonium formate. 

MS parameters were set as followed: capillary voltage: ±3500 V, desolvation temperature: 350 °C, desolvation flow: 650 L/h, and cone gas flow: 50 L/h. Cone voltage and collision energy were optimized for each analyte.

A Quality Control (QC) sample was used throughout the analytical batches. QC samples were prepared by mixing equal volumes of all tested samples, for the respective analysis, either syrup or powder.. QC samples were analyzed 5 times at the beginning of the analytical batch, for system equilibration. Also, a standard mixture containing all analytes of interest was injected in the beginning of the analytical run. Indicative chromatograms of a real samples (syrup and powder products) were illustrated in Figure S1 (supplementary information). 

Regarding method validation, linearity of the method was determined using different calibration standards per analyte, ranging between 0.01–2 mg/L up to 5–95 mg/L, depending on the analyte. Intra-day precision ranged between 0.5–7% for syrup samples and between 0.4–4% for carob powder samples. 

### 2.4. Data Handling–Statistics

LC-MS/MS data were collected and processed using MassLynx^®^ (Waters, Milford, MA, USA), while peak integration was performed using TargetLynx^®^ v4.1.

Analytes for further statistical evaluation were selected based on the criteria of either existing in the 60% of the samples analyzed or presenting a relative standard deviation (RSD) < 30% in QC samples. 

The selection of the optimized sample preparation protocol for either powder or syrup carob samples was based on the number of extracted peaks, the total area of maxima, classification of metabolites, and standard deviation of replicates. Microsoft Excel tools were used for illustration of results, while SIMCA 13.0 (Umetrics, Umea, Sweden) was used for the constructed principal component analysis (PCA) score plot in Unit Variance (UV) scaling. 

## 3. Results and Discussion 

In total, two replicates of the 20 different prepared carob samples of each matrix were analysed and further assessed the worth of commonly used organic solvents and their respective aqueous mixtures in two extraction ratios (Wc/Vs), as well as in three pH values. The aim was to define the optimal protocol for the analysis of both matrices, in order to fully cover the extracted metabolites. Sample preparation, a fundamental process prior to the analysis, plays a crucial role in the quality of the obtained results and the robustness of the methodology [30]. Carob and carob products are considered challenging matrices due to the physicochemical characteristics of raw carob fruit and processed carob products. 

The criteria of the optimal Wc/Vs ratio for both analysed matrices were the total area (sum of peak areas) and the number of maxima (higher peak areas) [30]. Based on both criteria, for both carob products, the 1:2 Wc/Vs ratio was selected as it provided higher intensities and peak areas, as expected. Neither deterioration of the analytical system nor saturation of the detector (as possible obstacles) were observed for the dense extracts; thus, the last was chosen.

**Figure 2:**
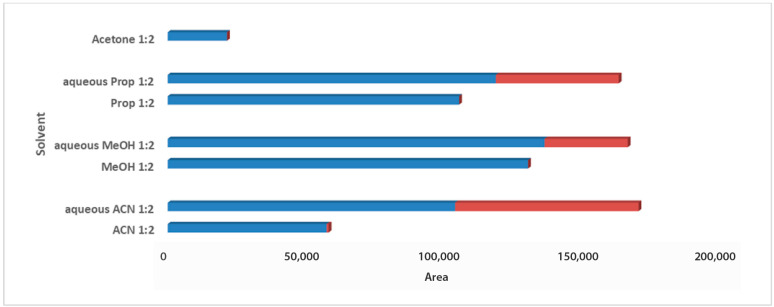
Bars plot of tested solvents and solvent mixtures in 1:2 Wc/Vs in syrup carob samples, indicated differences in total peak area and number of maxima (red colored).

**Figure 3:**
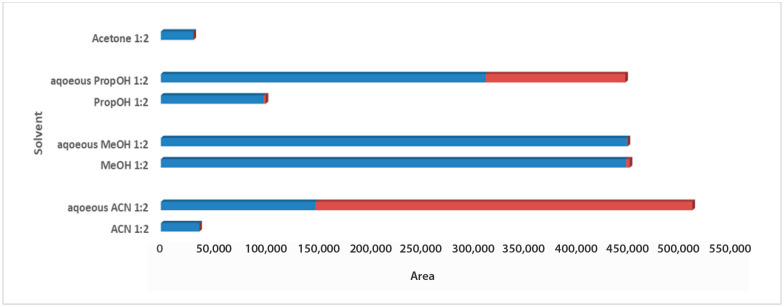
Bars plot of tested solvents and solvent mixtures in 1:2 Wc/Vs in powder carob samples, indicated differences in total peak area and number of maxima (red colored).

For the solvent selection in the 1:2 Wc/Vs ratio resulting from the previous step, acetone, ACN, MeOH, PropOH, ACN:H_2_O, MeOH:H_2_O, and PropOH:H_2_O were tested. The aforementioned solvents and solvent mixtures are easy to use, commonly available in analytical laboratories, less toxic, easy to evaporate, and suitable for one-step global analysis and for the extraction of polar and semi-polar analytes providing reproducible results. The aqueous organic solvents were evaluated to achieve enhanced extraction recovery of the metabolites. As illustrated in the bar plots for carob syrup (Figure 2), aqueous ACN 1:2 Wc/Vs presented the highest total area, followed by aqueous MeOH and aqueous PropOH, by an infinitesimal difference. The highest total peak area was also attributed to the highest number of maxima for aqueous ACN 1:2 Wc/Vs. Interestingly, while aqueous MeOH showed a higher total peak area compared to aqueous PropOH 1:2 Wc/Vs, the number of maxima presented the opposite trend. For carob powder, in Figure 3 it is observed that aqueous ACN 1:2 Wc/Vs presented the highest total peak area, as expected, due to the very large number of maxima. Although neat and aqueous MeOH 1:2 Wc/Vs and aqueous PropOH 1:2 Wc/Vs showed similar total peak areas, the aqueous PropOH 1:2 Wc/Vs was, notably, the only extraction solvent mixture that presented maximum peak areas. In both carob products, neat organic solvents indicated lower peak area values and a minor number of maximum peaks, compared to their respective aqueous mixtures. As expected, polar and semi-polar compounds were favorably extracted in the presence of an aqueous amount in the solvent mixture.

**Figure 4:**
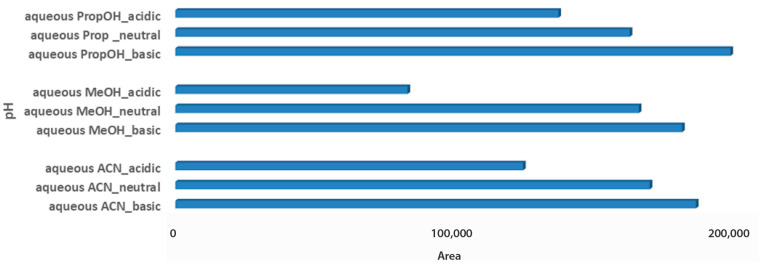
Bars plot of tested aqueous solvent mixtures in 1:2 Wc/Vs in different pH values (acid, neutral, basic), in syrup carob samples.

The last step for the sample optimization protocol was the pH evaluation in aqueous solvent mixtures. Carob is considered an acidic product, with a pH value close to 6.5, while the pH value of both tested products also ranged between 4.4 and 5.5 [32,33]. Thus, it was an interesting point to investigate, whereas pH adjustment of solvent mixtures would affect metabolites extraction of the acidic matrices. For syrup samples, independent of the aqueous extraction solvent, basic pH demonstrated higher total peak areas, as it probably favors the measured metabolites. Basic aqueous PropOH 1:2 Wc/Vs was the first choice based on both total peak area and number of metabolomic maxima (n = 14), followed by basic aqueous ACN 1:2 Wc/Vs (n = 7) and basic aqueous MeOH 1:2 Wc/Vs (n = 6). Neutral pH conditions for all tested aqueous mixture solvents illustrated comparable results (Figure 4). 

**Figure 5:**
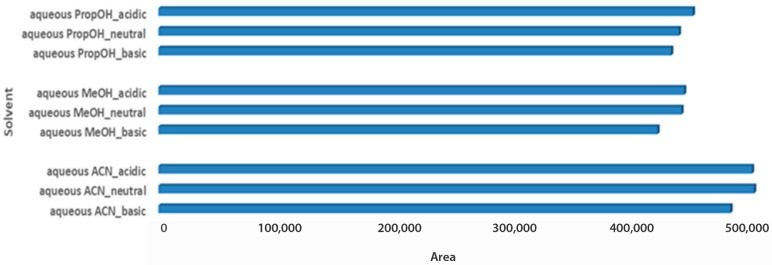
Bars plot of tested aqueous solvent mixtures in 1:2 Wc/Vs in different pH values (acid, neutral, basic), in powder carob samples.

**Figure 6:**
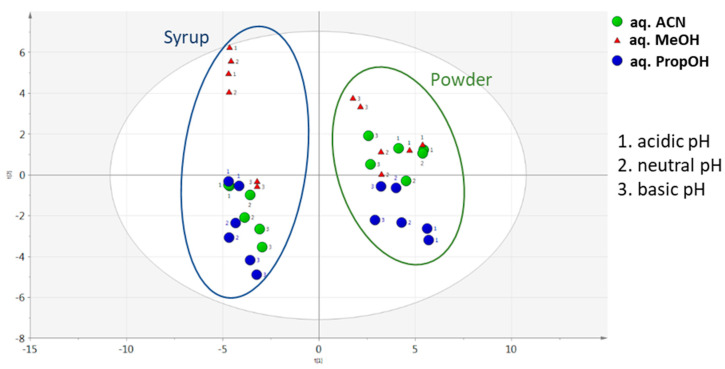
PCA score plot of carob products according to aqueous solvent mixtures and pH conditions.

In different pH conditions, aqueous ACN was the optimal extraction solvent mixture to extract carob powder metabolites for both assessed criteria (Figure 5). Infinitesimal differences were observed among different aqueous ACN pH conditions, with acidic having a slight predominance. A similar trend was also observed between aqueous PropOH and aqueous MeOH pH conditions, which showed satisfactory total peak areas. In the same manner, for aqueous MeOH, the number of maxima was not obtained. In the constructed PCA score plot a clear separation of the matrices was observed (Figure 6). Furthermore, the analysed samples were clustered by solvent mixture and propanol showed the lowest deviation in both matrices. Although a satisfying separation was observed, the effect of pH was milder compared to the solvent nature, as expected, since the solvent is determining factor in the sample preparation process. Validity of the constructed PCA scores plot model was based on the R2X and Q2 values. R2X was 0.731 and Q2 was 0.629, while CV-ANOVA was <0.05. 

From the 80 metabolites included in the targeted metabolomics-based method, the detected metabolites in each carob product were categorized into eight classes, according to their chemical taxonomy (Table 2), based on Human Metabolome Database (HMDB) [34]. 

In syrup, 32 compounds were not detected, while 16 metabolites namely adenine, creatine, creatinine, cytidine, kynurenate, maltose, nicotinic acid, putrescine, pyruvic acid, serine, taurine, theobromine, threonine, tyrosine, xanthine, and *γ*-aminobutyric acid, did not satisfy the aforementioned criteria. The highest number of compounds were met in 5 tested conditions, namely basic aqueous PropOH, PropOH, MeOH, neutral aqueous MeOH, and neutral aqueous ACN, while the lowest was met in ACN. The majority of the extracted analytes belonged to organooxygen compounds carbohydrates and carbohydrate conjugates, followed by carboxyl acids and derivatives, amino acids, peptides, and analogs, both classes of high biological significance. Notably, cytidine was only extracted in neutral aqueous ACN protocol, while nicotinic acid was favorably extracted in neutral aqueous PropOH. Caffeine was not detected since carob is a non-caffeine product [6]. To our surprise, glycine, isoleucine, arginine, tryptophan, aspartic acid, methionine, glutamic acid, lysine, and histidine were not detected or were under the limit of detection (LOD) for the applied method, although it was expected to be present in carob syrup. A possible explanation may be attributed to the thermal process of carob syrup production [35]. The total numbers of detected metabolites in syrup, based on the criteria described in Section 2.4, is summarized in Table 3.

A greater number of compounds were detected in carob powder samples compared to syrup. Forty-three out of 80 analytes were excluded according to the discussed criteria. Neutral aqueous ACN favored the extraction of most analytes, while neat ACN solvent extracted the least. Guanine was only extracted with aqueous PropOH but was further excluded from statistical evaluation. As illustrated in Table 4, most of the extracted analytes belonged to the same chemical categorization. 

This study attempted to provide an optimal sample preparation protocol for carob products. However, a single extraction sample preparation process for such diverse molecules with different physicochemical properties, is considered a challenging task. All criteria selected for the optimal conditions, number of extracted peaks, number of maxima, total area, and standard deviation of replicates, were chosen to provide a more global and, at the same time, selective approach limited for the specific carob analytes. 

Solvent evaporation and reconstitution in the mobile phase in the sample preparation protocol were performed to secure comparable results among the studied methodologies. The optimal extraction sample weight to solvent volume ratio was selected upon the set criteria, although the high density of the obtained extract could affect the analytical system’s performance (detector saturation, analytical column, source contamination, peak overlap). In an analysis of a large number of samples this should be taken into consideration. 

## 4. Conclusions

Carob syrup and carob powder sample preparation were studied for the optimal extraction of nutrient polar and semi-polar metabolites using HILIC-MS/MS. To our knowledge, this is the first attempt where various parameters, namely the number of metabolites, the effects of extraction, pH adjustment, solvent, and sample weight to solvent volume ratio (Wc/Vs) were examined with the aim of polar profiling the two different carob matrices. Aqueous acetonitrile at 1:2 Wc/Vs satisfied the established criteria for both carob syrup and powder samples. Nevertheless, when the pH was modified, a ratio of 1:2 Wc/Vs in basic pH using aqueous PropOH as the extraction solvent presented the optimal results for syrup analysis, while for carob powder analysis, acidic aqueous acetonitrile 1:2 Wc/Vs would be the best possible choice to obtain a satisfactory number of metabolites and extraction recovery. 

The current study suggests an optimal sample preparation protocol focused on small polar and semi-polar metabolites appropriate for HILIC-MS/MS analysis. The optimal process should be adapted to the specific needs and intentions of each study; thus, the suggested protocol should not be considered a universal approach. The optimal parameters offered either a global approach or a more selective one, for the extraction of specific metabolites. 

## Figures and Tables

**Table 2:** Chemical categorization for all detected analytes in carob syrup and carob powder.

Analyte	Chemical Category	Matrix
Betaine	carboxyl acids + deriv./amino acids, peptides + analogues	S *, P **
*γ*-aminobutyric acid	carboxyl acids + deriv./amino acids, peptides + analogues	P
Pyroglutamic acid	carboxyl acids + deriv./amino acids, peptides + analogues	S, P
Cellobiose	organooxygen compounds/carbohydrates + carbohydrate conjugates	S, P
Thiamine	diazines/pyrimidines, pyrimidines derivatives	P
Proline	carboxyl acids + deriv./amino acids, peptides + analogues	S, P
Maltose	organooxygen compounds/carbohydrates + carbohydrate conjugates	P
Sorbitol	organooxygen compounds/carbohydrates + carbohydrate conjugates	S, P
Sucrose	organooxygen compounds/carbohydrates + carbohydrate conjugates	S, P
Lactose	organooxygen compounds/carbohydrates + carbohydrate conjugates	S, P
Trehalose	organooxygen compounds/carbohydrates + carbohydrate conjugates	S, P
Theobromine	imidazonepyrimidines/purines	P
Melibiose	organooxygen compounds/carbohydrates + carbohydrate conjugates	S, P
Benzoic acid	Benzene + substitutes derivatives/benzoic acids + deriv.	S, P
Alanine	carboxyl acids + deriv./amino acids, peptides + analogues	S, P
Leucine	carboxyl acids + deriv./amino acids, peptides + analogues	S, P
Phenylalanine	carboxyl acids + deriv./amino acids, peptides + analogues	S, P
Valine	carboxyl acids + deriv./amino acids, peptides + analogues	S, P
Lactic acid	hydroxy acids + deriv./a-hydroxyacids + deriv.	S, P
Monoisoamylamine	organonitrogen compounds/amines	S, P
Choline	organonitrogen compounds/quaternary ammonium salts	S, P
Nicotinamide	pyridines + deriv./Pyridinecarboxylic acids and derivatives	S, P
Nicotinic acid	pyridines + deriv./Pyridinecarboxylic acids and derivatives	S, P
Uridine	pyrimidine nucleosides	S, P
Guanine	imidazonepyrimidines/purines	P
Fructose	organooxygen compounds/carbohydrates + carbohydrate conjugates	S, P
Glucose	organooxygen compounds/carbohydrates + carbohydrate conjugates	S, P
Adenosine	purine nucleosides	P
Pyridoxine	Pyridines +derivat./Pyridinecarboxylic acids and derivatives	S, P
Acetyl-carnitine	fatty acyls/fatty acids + conjugates	S, P
*α*-Hydroxyisovaleric acid	fatty acyls/fatty acids + conjugates	S, P
Itaconic acid	fatty acyls/fatty acids + conjugates	P
Hypoxanthine	imidazonepyrimidines/purines	S, P
Xanthine	imidazonepyrimidines/purines	P
Ribose	organooxygen compounds/carbohydrates + carbohydrate conjugates	S, P
Xylose	organooxygen compounds/carbohydrates + carbohydrate conjugates	S, P
Ascorbic acid	dehydrofuranes/furanones	S
Uracil	diazines/pyrimidines, pyrimidines derivatives	S
Mannose	organooxygen compounds/carbohydrates + carbohydrate conjugates	S, P
Cotinine	Pyridines + deriv./pyrrolidinylpyridines	S
Cytidine	pyrimidine nucleosides	S
Thymidine	pyrimidine nucleosides	S

* S: Carob syrup; ** P: Carob powder.

**Table 3:** Number and chemical categorization of compounds extracted upon the applied conditions in carob syrup.

Chemical Class/Subclass	aq. PropOH Acid	aq. PropOH Neutral	aq. PropOH Basic	PropOH	aq. MeOH Acid	aq. MeOH Neutral	aq. MeOH Basic	MeOH	aq. ACN Acid	aq. ACN Neutral	aq. ACN Basic	ACN
**CARBOXYL ACIDS + DERIV./AMINO ACIDS, PEPTIDES + ANALOGS (**n **= 7)**	7	7	7	7	6	7	7	7	7	7	7	4
**FATTY ACYLS/FATTY ACIDS ESTERS (**n **= 2)**	2	2	2	2	2	2	2	2	2	2	2	1
**IMIDAZONEPYRIMIDINES/PURINES (**n **= 1)**	1	1	1	1	1	1	1	1	1	1	1	0
**ORGANONITROGEN COMPOUNDS/AMINES (**n **= 2)**	2	2	2	2	2	2	2	2	2	2	2	1
**ORGANOOXYGEN COMPOUNDS/CARBOHYDRATES + CARBOHYDRATE CONJUGATES (**n **= 11)**	10	10	11	11	10	11	11	11	11	11	11	9
**PYRIDINES + DERIV./PYRIDINE CARBOXYLIC ACIDS AND DERIVATIVES (**n **= 4)**	2	4	3	3	3	3	2	3	2	3	2	3
**PYRIMIDINE NUCLEOSIDES (**n **= 3)**	2	2	2	2	2	2	2	2	2	3	2	2
**OTHERS (**n **= 4)**	4	4	4	4	4	4	4	4	4	4	4	3
**Total (N = 34)**	30	32	32	32	30	32	31	32	31	33	31	23

**Table 4:** Number and chemical categorization of compounds extracted upon the applied conditions in carob powder.

Chemical Class/Subclass	aq. ACN Basic	aq. ACN Acidic	aq. ACN Neutral	ACN	aq. MeOH Basic	aq. MeOH Acidic	aq. MeOH Neutral	MeOH	aq. PropOH Basic	aq. PropOH Acidic	aq. PropOH Neutral	PropOH
**CARBOXYL ACIDS + DERIV./AMINO ACIDS, PEPTIDES + ANALOGS (**n **= 8)**	7	8	8	6	8	7	8	8	7	8	8	7
**FATTY ACYLS/FATTY ACIDS + CONJUGATES (**n **= 3)**	2	3	3	1	2	1	3	3	3	3	3	2
**IMIDAZONEPYRIMIDINES/PURINES (**n **= 4)**	3	3	4	1	3	3	3	3	4	4	4	2
**ORGANONITROGEN COMPOUNDS/AMINES (**n **= 2)**	2	2	2	1	2	2	2	2	2	2	2	2
**ORGANOOXYGEN COMPOUNDS/CARBOHYDRATESCARBOHYDRATE CONJUGATES (**n **= 12)**	12	12	12	9	11	12	12	12	12	11	12	11
**PYRIDINES + DERIV./PYRIDINE CARBOXYLIC ACIDS AND DERIVATIVES (**n **= 3)**	3	3	3	2	3	3	3	3	3	3	3	2
**PYRIMIDINE NUCLEOSIDES (**n **= 1)**	1	1	1	1	1	1	1	1	1	1	1	1
OTHERS (n = 4)	4	4	4	3	4	4	4	4	3	4	3	3
**Total (n = 37)**	34	36	37	24	34	33	36	36	35	36	36	30

## Data Availability

The data presented in this study are available on request from the corresponding author. The data are not publicly available due to ongoing research.

## Supplementary Materials

The following supporting information can be downloaded at: https://www.mdpi.com/article/10.3390/metabo13050645/s1, Figure S1: Chromatograms of uridine, nicotinamide, trehalose, choline, betaine and benzoic acid of powder (A) and a syrup (B) products, after the extraction with the respective optimal conditions.
