# Supplementary material for: Optimization of Carob Products Preparation for Targeted LC-MS/MS Metabolomics Analysis
Source: Metabolites. 2023 May 9;13(5):645. doi: 10.3390/metabo13050645 (PMC10224476; doi:10.3390/metabo13050645)
Supplement: Supplementary file 1 [file metabolites-13-00645-s001.zip › metabolites-2346081-supplementary.pdf]

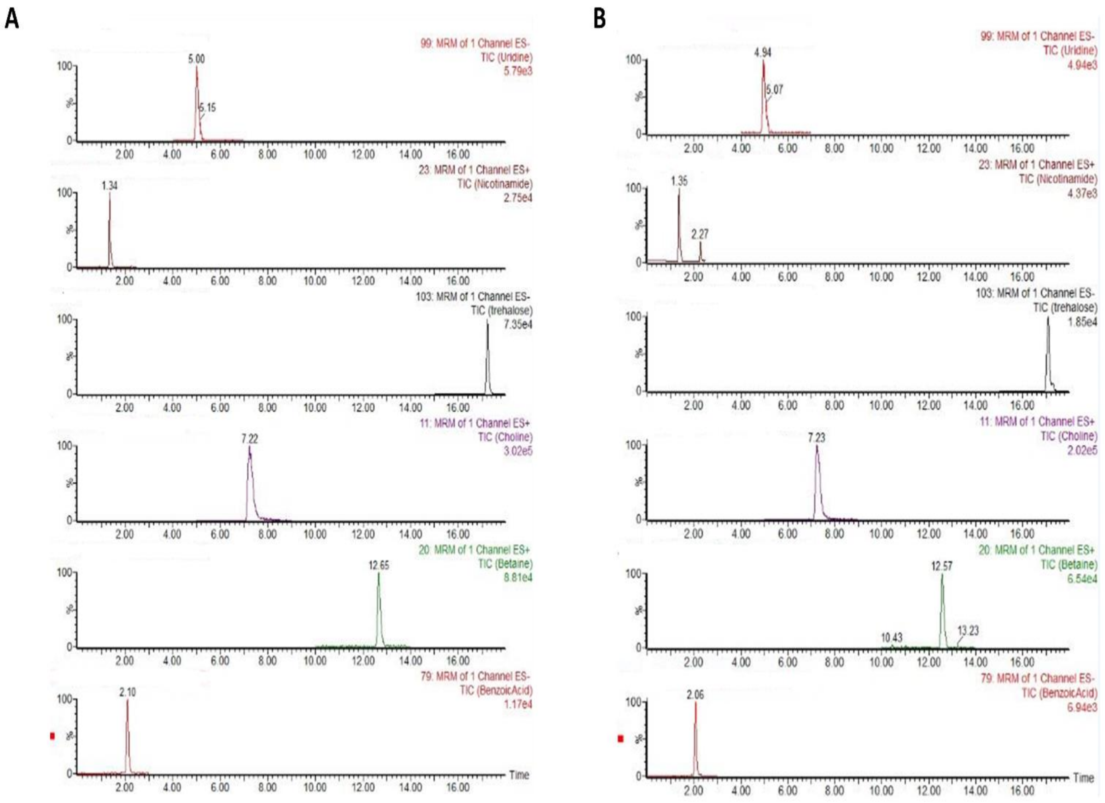

Figure S1: Chromatograms of uridine, nicotinamide, trehalose, choline, betaine and benzoic acid of powder (A) and a syrup (B) products, after the extraction with the respective optimal conditions.
